# Sudden spleen rupture in a *Plasmodium vivax*-infected patient undergoing malaria treatment

**DOI:** 10.1186/s12936-018-2228-2

**Published:** 2018-02-13

**Authors:** Aleix Elizalde-Torrent, Fernando Val, Ingrid Cardoso C. Azevedo, Wuelton M. Monteiro, Luiz C. L. Ferreira, Carmen Fernández-Becerra, Hernando A. del Portillo, Marcus V. G. Lacerda

**Affiliations:** 1 ISGlobal, Hospital Clínic, Universitat de Barcelona, Barcelona, Spain; 2 Fundação de Medicina Tropical Dr. Heitor Vieira Dourado (FMT-HVD), Manaus, Amazonas Brazil; 3 Universidade do Estado do Amazonas (UEA), Manaus, Amazonas Brazil; 4 Institut d’Investigació Germans Trias i Pujol (IGTP), Badalona, Spain; 5 Institució Catalana de Recerca i Estudis Avançats (ICREA), Barcelona, Spain; 6 Fundação Oswaldo Cruz, Instituto Leônidas e Maria Deane (FIOCRUZ-Amazonas), Manaus, Amazonas Brazil

**Keywords:** *Plasmodium vivax*, Malaria, Spleen rupture, Portable ultrasound, Recurrent parasitemia, Splenectomy

## Abstract

**Background:**

Splenomegaly is one of the most common features of malaria. However, spontaneous splenic rupture, although unusual, represents a severe complication often leading to death. It is mostly seen in acute infection and primary attack, and it is most commonly associated with *Plasmodium vivax*. Here, a case of spontaneous splenic rupture diagnosed with a portable ultrasound apparatus shortly after starting treatment and with recurrent parasitaemia after splenectomy, is reported.

**Case description:**

In November 2015, a 45-year-old Brazilian man presented to the hospital in Manaus with fever, headache and myalgia. He was diagnosed with *P. vivax* malaria and, after a normal G6PD test, he started treatment with chloroquine and primaquine and was discharged. Two days later, he went back to the hospital with abdominal pain, dyspnea, dry cough, pallor, oliguria and fever. Using a portable ultrasound, he was diagnosed of rupture of the spleen, which was removed by emergency surgery. After this episode, he suffered two more malaria episodes with high parasitaemia at approximately 2-month intervals. DNA from different portions of the spleen was extracted and a qualitative PCR was performed to detect *P. vivax*.

**Conclusions:**

The splenic rupture suffered by this patient occurred 2 days after starting the treatment. Having a portable ultrasound apparatus may have saved the patient’s life, as it revealed a haemorrhage needing an urgent surgery. Parasites were detected by PCR in the extracted spleen. This patient suffered two more vivax malaria diagnosed episodes in spite of receiving and completing treatment with chloroquine and primaquine for each clinical attack. Splenic rupture during acute malaria is uncommon, but it is likely underdiagnosed and underreported, because the lack of means and equipment hinders diagnostic confirmation, especially in endemic areas.

## Background

Splenic rupture has a traumatic origin in most cases. Other cases occur in a pathological spleen, with haematological malignancy being the leading cause, followed by infections, and vascular, genetic, and haematological disorders [1]. However, in malaria-endemic countries, infection outnumbers haematological malignancies. Splenic rupture is more prevalent among infections caused by *Plasmodium vivax* than other species, although precise data on its incidence is presently unknown [2–4]. Malaria constitutional symptoms preceding splenic rupture may be diverse, and can even remain almost asymptomatic. Common symptoms comprise abdominal pain, including tenderness and rigidity in the left upper quadrant, which can radiate to the left shoulder (Kehr’s sign), enlarged spleen, fever, tachycardia, haemodynamic collapse, nausea and vomiting. Splenic rupture due to malaria is a concerning issue because, even with good treatment strategies and rapid therapy initiation, death rates are known to be around 38% in travelers and about 10% in indigenous people from malaria endemic regions [3]. In addition, there are still issues regarding opportune recognition and management in such cases.

## Case presentation

A 45-year-old Brazilian man from Manaus, Western Brazilian Amazon, presented to the hospital of the Fundação de Medicina Tropical Doutor Heitor Vieira Dourado with fever, headache, and myalgia during the previous 5 days. Physical examination showed no alterations. He was diagnosed with *P. vivax* malaria (parasitaemia of 0.26%), and, after a normal qualitative G6PD test, treatment with chloroquine and primaquine were prescribed following the national guidelines [for this patient, chloroquine for 3 days (600 mg the first, 450 mg the second and 450 mg the third day) and primaquine for 8 days (30 mg/day)].

The patient was discharged, but 2 days later, he returned to the hospital with abdominal pain, dyspnea, dry cough, oliguria, and fever, showing hypotension, tachycardia, pallor, and sweating on arrival. Abdominal examination revealed tenderness in the left hypochondriac region, rebound tenderness and abdominal guarding. A blood film showed a *P. vivax* parasitaemia of 0.006%. Blood analysis revealed 5.53 g/dL of haemoglobin, 15.98% of haematocrit, 22,000 platelets/mm^3^, 1.59 mg/dL of total bilirubin, 1.48 mg/dL of indirect bilirubin, 3.1 mg/dL of creatinine and INR of 1.43. Despite the low parasitaemia, severe malaria was considered as a result of the combination of clinical and laboratory data. No other laboratory tests were done to rule out different infectious causes. The patient received a blood transfusion and primaquine was suspended because of thrombocytopaenia. At this moment, due to a suspicion of spleen rupture and patient instability, a portable ultrasound scan was performed. This revealed a layer of free fluid in the splenorenal space and in the pelvis. The patient was referred to a surgery hospital (Hospital Pronto Socorro 28 de Agosto, also in Manaus), where he underwent an emergency splenectomy. Ten days after surgery, the patient had a positive blood smear (0.007%), anaemia and thrombocytopaenia. A negative blood smear was obtained 14 days after abdominal pain appeared. It was only a month after the splenectomy that blood parameters began to ameliorate, and primaquine was re-started at this point to attempt clearing of hypnozoites. The dose of primaquine given was 30 mg/day for 8 days, representing a total dose of 3.2 mg/kg.

Two months after the beginning of the symptoms and 1 month after reintroducing primaquine, the patient had another malaria episode. He presented with fever, headache and myalgia for 1 day, and parasitaemia was 0.32%. He received a new chloroquine + primaquine treatment cycle. Seven days later, the patient was asymptomatic and the blood smear negative. The dose of primaquine was the same as the previous time, although treatment was not supervised. However, 2 months after the latest episode, the patient suffered another malaria attack (1.3% parasitaemia), and he received the same treatment one more time, although again treatment was not supervised. No features of severe malaria were present in either of the two episodes. Patient follow-up after the last episode was not possible as he moved out of Manaus.

**Fig. 1:**
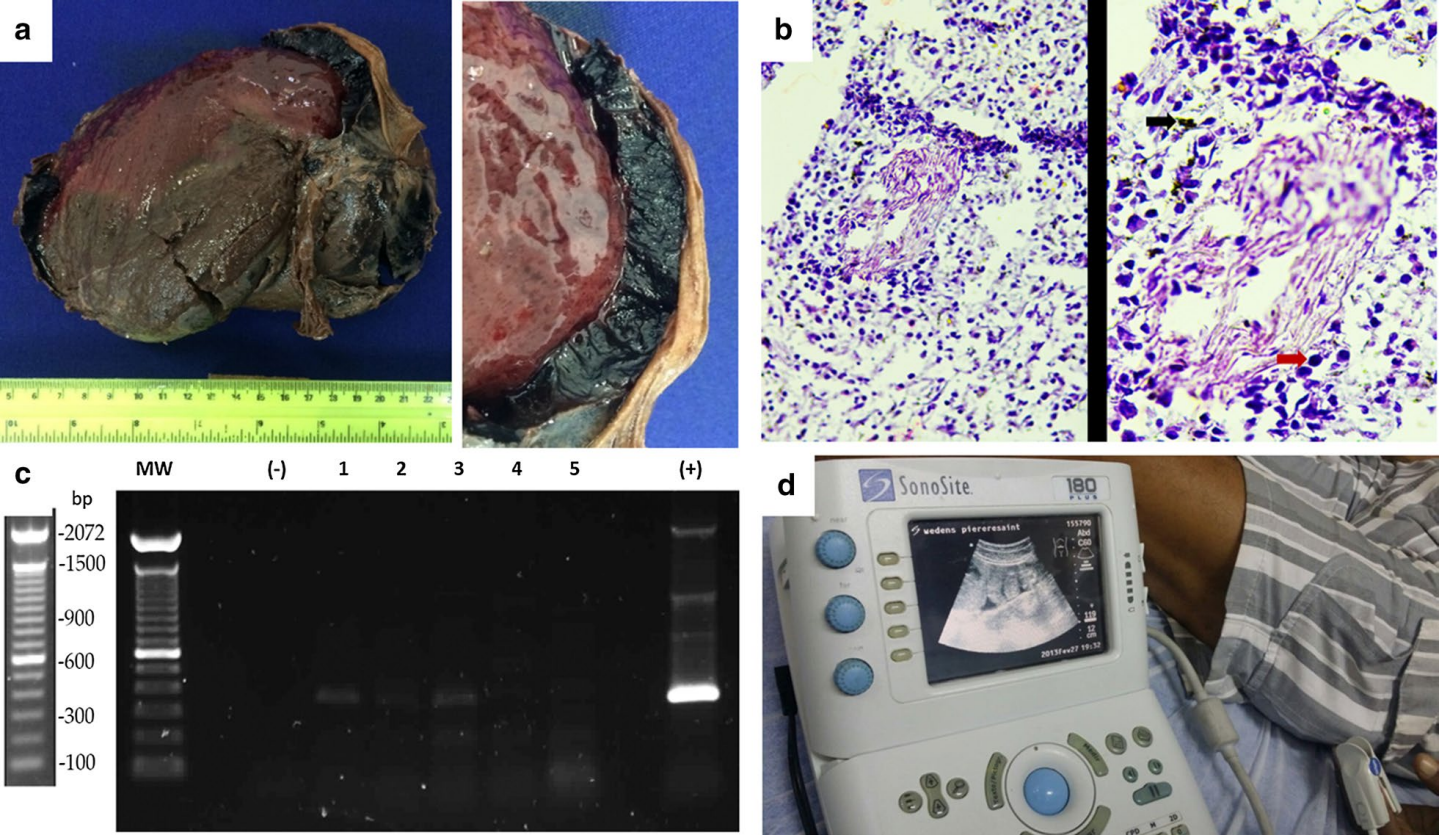
Spleen rupture analysis and diagnostics. **a** The enlarged and lacerated spleen with a visible subcapsular hematoma after removal. **b** Optical microscopy images of the spleen after haematoxylin–eosin stain at increasing magnification. A red pulp cord infiltration by lymphoid cells (red arrow) and remnants of parasite’s haemozoin (black arrow) can be observed, similar to a previous report [15]. **c** Qualitative PCR for *P. vivax*-MSP_1(19)_ detection in five portions. **d** Portable ultrasound machine used in confirming the spleen rupture

The patient gave consent to analyse the removed spleen at the time of splenectomy. Upon visual inspection, the spleen appeared enlarged and displayed multiple lacerations. After being immersed in a fixative solution, examination of the ruptured spleen showed areas of different coloration and consistency (Fig. 1a). Very solid brownish areas, some more liquid reddish ones and an almost black, large subcapsular haematoma could be distinguished. The size of the organ was 17 × 14 × 7.5 cm. Splenic sections were embedded in OCT (Optimal Cutting Temperature compound) and frozen for histopathology analysis 6 months later. This analysis revealed red pulp cord infiltration by lymphoid cells and remnants of parasite’s haemozoin (Fig. 1b). In addition, DNA from different spleen portions was extracted, and a PCR using primers for the *P. vivax*-MSP_1(19)_ (merozoite surface protein) gene, showed the presence of the parasite (Fig. 1c).

## Discussion

*Plasmodium vivax* is the most widely distributed human malaria parasite with an at-risk population of 2.5 billion [5]. In 2015 it caused approximately 8.5 million cases, of which 58% occurred in the WHO South-East Asia Region and 3000 deaths [6]. Contrary to what was believed some years ago, molecular diagnosis techniques, and ruling out other infections or chronic diseases, have demonstrated that the *P. vivax* mono-infection is involved in multiple-organ dysfunction and severe life-threatening disease. *Plasmodium vivax* infection can cause severe anaemia, hepatic dysfunction and jaundice, acute lung injury, acute respiratory distress syndrome and pulmonary oedema, shock, acute renal failure, severe thrombocytopaenia and splenic rupture [7, 8]. It is still unclear if cerebral malaria is also among clinical manifestations [9]. Patients with no or low immunity to malaria have the highest risk of spleen rupture [10]. Therefore, patients with acute malaria should avoid activities with risk of abdominal trauma during several weeks after recovering from the acute disease [10].

In the present case, the patient had recurrent parasitaemia before and after the surgery, and suffered two more malaria attacks after the spleen rupture. Unfortunately, at present there are no reliable biomarkers capable of differentiating malaria episodes originating from recrudescence, reinfection or relapse. Yet, it is likely that the recurrences in this case are indeed due to relapses, since the patient remained within the urban area of Manaus during the study period. Because treatment was not supervised and since reports of malaria resistant to primaquine are generally related to deficient supervision [11], inadequate primaquine dosing could be a plausible explanation for the recurrences observed in this patient. Also, low activity of the patient’s CYP2D6 enzyme should be considered as an alternative explanation for the recurrences observed (analysis not performed) [12]. Of note, this patient presented increasing parasitaemia at each attack, supporting the important role that the spleen plays in malaria [3, 13, 14].

The spleen could be a niche for *P. vivax* leading to splenomegaly and maintenance of a low peripheral parasitaemia. Supporting this hypothesis, a spleen rupture previously reported from Manaus in a non-immune patient with *P. vivax* monoinfection who was splenectomized 2 days before diagnosis and treatment of infection, showed intact *P. vivax*-infected reticulocytes in the cords [15]. Unfortunately, all attempts to detect intact parasites in the current spleen by immunofluorescence analysis were unsuccessful. Yet, histopathological analysis showed equivalent findings, and PCR demonstrated the presence of *P. vivax* DNA in this spleen.

There are several unknowns on splenic rupture yet to be clarified. The underlying pathophysiology of the spleen rupture has not been fully unveiled. However, two mechanisms, both occurring in acute malaria, have been involved in the process. The first is an effect of activated lymphatic tissue of the spleen and marked stasis in its sinuses produced by deformed infected red blood cells with altered surface characteristics [10]. The second mechanism is a consequence of spleen compression by abdominal musculature during physiological activities such as sneezing, coughing, defecation and sitting up or turning in bed [2].

Another matter to be emphasized is about splenic rupture occurring in acute versus chronic malaria. In acute malaria, the spleen is soft and its capsule is thin and tightly stretched. Rupture may, therefore, occur with minor trauma or spontaneously, and is primarily single staged, with haematoma formation and rupture nearly occurring at the same time. In contrast, splenic rupture in chronic malaria is infrequent, and when it occurs, the spleen normally ruptures in two stages: an initial subcapsular haematoma progresses, which later ruptures through the capsule days to weeks later. It is usually preceded by an obvious trauma. Regarding this observation, a thick splenic capsule detected in patients with chronic malaria and schistosomiasis may delay rupture of the subcapsular haematoma [4]. Related to this topic, patients with low immunity or no immunity to malaria have the highest risk of rupture. Thus, an abnormal immunological response during the acute stage causes a rapid growth of the spleen, which drives to a higher incidence of splenic rupture. Therefore, all patients with acute malaria should be advised to avoid activities with increased risk of abdominal trauma for several weeks after curing acute disease [10].

Concerning the diagnosis, and from the clinical point of view, it is important to note that the longer the symptoms of malaria attacks persist, the higher the risk for splenic rupture, with early diagnosis becoming of utmost importance for prevention [3]. In that sense, splenic rupture should always be suspected in a patient from an endemic region displaying a rapid onset of hypotension and left hypochondrial pain [2]. Confirmation of the splenic rupture in a haemodynamically stable patient can be obtained by ultrasonography, computerized tomography scanning, and arteriography. Computerized tomography has replaced angiography as the preferred diagnostic tool, and is useful in diagnosis and monitoring a patient in whom conservative management of splenic rupture is considered [2]. Yet, in many endemic regions, computerized tomography is not economically feasible and often not available. By contrast, ultrasound technology can be more easily available economically and geographically. In the clinical case presented here, a portable ultrasound apparatus used at the proper moment by a competent professional may have most probably saved the patient’s life (Fig. 1d).

Portable ultrasound devices are widely used in medicine, as operation of ultrasounds is non-invasive, inexpensive and not associated with radiation exposure [16]. Although they are primarily used for diagnostic purposes, they are also used for therapeutic intentions (e.g. guided punctures). They have shown utility in limited resource settings and in remote areas where treatment may be improved by transmitting images to central sites [17]. The Fundação de Medicina Tropical is a reference hospital for infectious diseases in Manaus. Most of the medical staff in the emergency department are clinicians (general practitioners, pediatricians, infectiologists, and dermatologists). There is also a radiology department, although radiologists are not present 24 h a day. Minor interventions of elective surgery can be performed in the hospital. However, when this patient arrived, there was no radiologist nor surgeon in the hospital. The clinician did the ultrasonography and, after that, the patient was referred to the general hospital. There, another portable ultrasound scanner was used and surgery started in a short period.

In terms of treatment, non-operative management can be used in carefully selected cases [3, 4]. Transcatheter splenic artery embolization can be performed in selective cases of non-traumatic rupture [18]. But, in the case of a life threatening situation with uncontrolled bleeding and haemorrhagic shock, splenectomy is still the treatment of choice [4]. In case of operative management, spleen-preserving procedures should be, whenever possible, the standard, especially in patients with a high probability of future exposure to malaria [19–22].

## Conclusion

In conclusion, splenic rupture during acute malaria is rare but is likely underdiagnosed and underreported. It is a life-threatening malaria complication that can occur after starting treatment [3]. Therefore, early diagnosis and appropriate disease management are essential [18]. In the clinical case presented here, the availability and use of a portable ultrasound apparatus may have saved the patient’s life.

## References

[1] Orloff MJ, Peskin GW (1958). Spontaneous rupture of the normal spleen; a surgical enigma. Int Abstr Surg.

[2] Hussein BMA, Al Ani AM, Al-Mayoofi O, Mehraj M, Joher AA, Bonilla JA (2016). Spontaneous rupture of splenic hematoma in a malaria patient: case report and review of literature. Int J Surg Case Rep.

[3] Imbert P, Rapp C, Buffet PA (2009). Pathological rupture of the spleen in malaria: analysis of 55 cases (1958–2008). Travel Med Infect Dis.

[4] Hamel CT, Blum J, Harder F, Kocher T (2002). Nonoperative treatment of splenic rupture in malaria tropica: review of literature and case report. Acta Trop.

[5] Howes RE, Battle KE, Mendis KN, Smith DL, Cibulskis RE, Baird JK (2016). Global epidemiology of *Plasmodium vivax*. Am J Trop Med Hyg.

[6] WHO (2017). World malaria report.

[7] Price RN, Tjitra E, Guerra CA, Yeung S, White NJ, Anstey NM (2007). Vivax malaria: neglected and not benign. Am J Trop Med Hyg.

[8] Lacerda MVG, Fragoso SCP, Alecrim MGC, Alexandre MAA, Magalhães BML, Siqueira AM (2012). Postmortem characterization of patients with clinical diagnosis of *Plasmodium vivax* malaria: to what extent does this parasite kill?. Clin Infect Dis.

[9] Lampah DA, Yeo TW, Hardianto SO, Tjitra E, Kenangalem E, Sugiarto P (2011). Coma associated with microscopy-diagnosed *Plasmodium vivax*: a prospective study in Papua, Indonesia. PLoS Negl Trop Dis.

[10] Gockel HR, Heidemann J, Lorenz D, Gockel I (2006). Spontaneous splenic rupture, in tertian malaria. Infection.

[11] Baird JK, Hoffman SL (2004). Primaquine therapy for malaria. Clin Infect Dis.

[12] Bennett JW, Pybus BS, Yadava A, Tosh D, Sousa JC, McCarthy WF (2013). Primaquine failure and cytochrome P-450 2D6 in *Plasmodium vivax* malaria. N Engl J Med.

[13] Baird JK (2013). Evidence and implications of mortality associated with acute *Plasmodium vivax* malaria. Clin Microbiol Rev.

[14] Barber BE, William T, Grigg MJ, Parameswaran U, Piera KA, Price RN (2015). Parasite biomass-related inflammation, endothelial activation, microvascular dysfunction and disease severity in vivax malaria. PLoS Pathog.

[15] Machado Siqueira A, Lopes Magalhães BM, Cardoso Melo G, Ferrer M, Castillo P, Martin-Jaular L (2012). Spleen rupture in a case of untreated *Plasmodium vivax* infection. PLoS Negl Trop Dis..

[16] CADTH Rapid Response Reports. Portable ultrasound devices in the pre-hospital setting: a review of clinical and cost-effectiveness and guidelines. Ottawa: Canadia Agency for Drugs and Technologies in Health; 2015. 26985544

[17] El Sayed MJ, Zaghrini E (2013). Prehospital emergency ultrasound: a review of current clinical applications, challenges, and future implications. Emerg Med Int.

[18] Kim NH, Lee KH, Jeon YS, Cho SG, Kim JH (2015). Spontaneous splenic rupture in a vivax malaria case treated with transcatheter coil embolization of the splenic artery. Korean J Parasitol.

[19] de Aguirre Z, De Droogh E, Van den Ende J, Lynen L, De Praetere K, Demey HE (1998). Splenic rupture as a complication of *P. falciparum* malaria after residence in the tropics. Report of two cases. Acta Clin Belg.

[20] Mabogunje OA (1990). Conservation of the ruptured spleen in children: an African series. Ann Trop Paediatr.

[21] Rapp C, Debord T, Imbert P, Lambotte O, Roué R (2002). Splenic rupture in infectious disease: splenectomy or conservative treatment? Report of three cases. Rev Med Interne.

[22] Zingman BS, Viner BL (1993). Splenic complications in malaria: case report and review. Clin Infect Dis.

